# Solid-Phase Synthesis of a New Diphosphate 5-Aminoimidazole-4-carboxamide Riboside (AICAR) Derivative and Studies toward Cyclic AICAR Diphosphate Ribose

**DOI:** 10.3390/molecules16098110

**Published:** 2011-09-21

**Authors:** Stefano D’Errico, Giorgia Oliviero, Nicola Borbone, Jussara Amato, Vincenzo Piccialli, Michela Varra, Luciano Mayol, Gennaro Piccialli

**Affiliations:** 1 Dipartimento di Chimica delle Sostanze Naturali, Università degli Studi di Napoli Federico II, Via D. Montesano, 49, 80131, Napoli, Italy; 2 Facoltà di Scienze Biotecnologiche, Università degli Studi di Napoli Federico II, Via D. Montesano, 49, 80131, Napoli, Italy; 3 Dipartimento di Chimica Organica e Biochimica, Università degli Studi di Napoli Federico II, Via Cynthia, 4, 80126, Napoli, Italy

**Keywords:** AICAR, cADPR, solid-phase synthesis

## Abstract

The solid-phase synthesis of the first example of a new diphosphate AICAR derivative is reported. The new substance is characterized by the presence of a 5'-phosphate group while a second phosphate moiety is installed on a 5-hydroxypentyl chain attached to the 4-*N*-position of AICAR. Cyclization of the diphosphate derivative by pyrophosphate bond formation allowed for the formation of a novel AICAR-based cyclic ADP-ribose (cADPR) mimic.

## 1. Introduction

Modified nucleosides and nucleotides are among the most studied biomolecules due to their numerous biological and pharmacological applications. Apart from being the genomic building blocks, nucleosides interact with roughly one-third of the protein classes in the human genome, including polymerases, kinases, reductases, motor proteins, membrane receptors, and structural proteins, all targets of biological importance.

**Figure 1:**
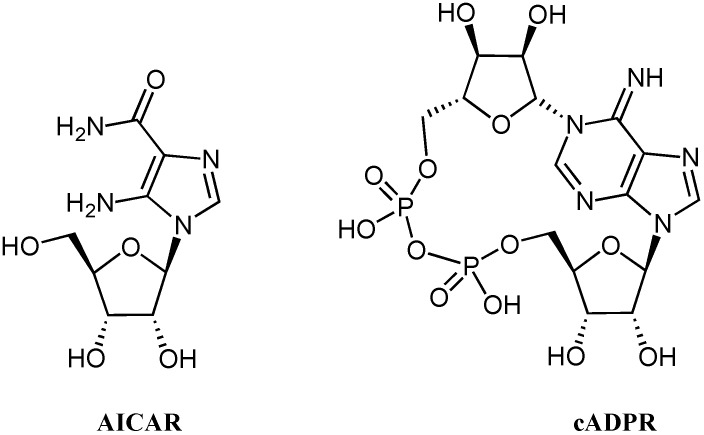
Structures of AICAR and cADPR.

Many nucleoside analogues are currently used as antiviral [1,2], antineoplastic [3,4,5,6] and antifungal [7,8,9] agents. Numerous nucleoside derivatives interact with enzymes such as CD38 [10] or 5'-AMP-activated protein kinase (AMPK), playing a key role in the signal transduction or regulation of energy metabolism. In particular, 5-aminoimidazole-4-carboxamide riboside (AICAR, Figure 1) activates the AMPK pathway in skeletal muscle, adipocytes and hepatocytes [11,12,13]. The adipocytes and adipose tissue-derived macrophages release a variety of pro-inflammatory cytokines and chemokines including tumor necrosis factor alpha (TNF alpha), interleukin-6 and interleukin-8, the production of which is enhanced in the obese state [11,12,13]. Moreover, recent studies indicate that stimulation of the AMPK system by AICAR in type 2 diabetic patients reduces plasma glucose and NEFA (non-esterified fatty acids) [14]. Furthermore, AICAR has been indicated as a promising prodrug, which induces benefits in patients suffering from autism, cerebral palsy, insomnia, schizophrenia, and other neuropsychiatric symptoms generally associated to chronic low levels of adenosine [15].

However, AICAR has a short half-life in the cell [16], it does not efficiently cross the blood–brain barrier and is poorly adsorbed from the gastrointestinal tract. Consequently, the production of new AICAR derivatives is an appealing objective in the field of medicinal chemistry. In recent years, the syntheses of a number of AICAR derivatives, such as the 2-aryl-, 4*-N*-benzyl-, 4-substituted-, 5-substituted-, 5-hydroxyl- (bredinin), triazolyl-riboside- (ribavirin), and 2′,3′-secoriboside derivatives, have been reported in the literature [17]. AICAR is phosphorylated in the cell at the 5′-position to produce AICAR 5′-monophosphate (ZMP) which in turn is expected to possess higher activity than AICAR in AMPK activation. On these grounds we decided to synthesise a new type of AICAR derivative embodying two phosphate groups using a solid-phase strategy recently developed in our laboratories to prepare ZMP [18] and some 4-*N*-alkyl AICA-ribotides [17].

Furthermore, the presence of two fully deprotected phosphate groups allowed us to prepare an unprecedented mimic of cyclic ADP-ribose (cADPR, Figure 1) in which 5-aminoimidazole-4-carboxamide was constructed instead of the adenine moiety and the northern ribose was replaced by a hydroxypentyl chain. The cADPR analogue was obtained by the cyclization of the corresponding linear precursor once detached from its respective solid support.

## 2. Results and Discussion

**Scheme 1:**
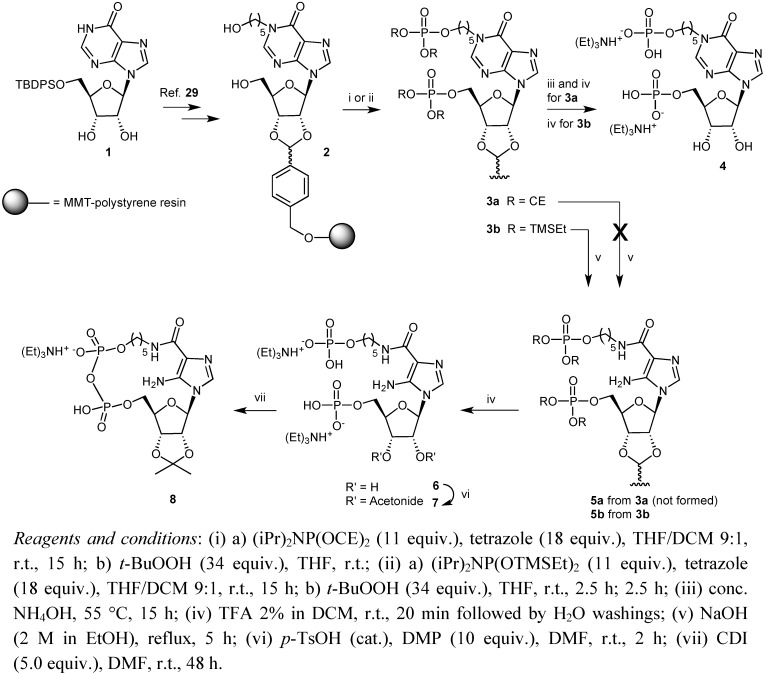
Preparation of compounds **4** and **6-8**.

In a previous study we described the solid-phase synthesis of a number of 4-*N*-alkyl derivatives of ZMP through the pyrimidine ring degradation of suitable 5'-phosphate inosine precursors [18]. As a further extension of this strategy, we report here the synthesis of the new diphosphate AICAR derivative **6** (Scheme 1) carrying phosphate groups at both the 5'-position and on a 5-hydroxypentyl chain bonded to the 4-*N*-position of AICAR.

The proposed solid-phase procedure can be easily extended to the preparation of further analogues of the same class simply by modulating the 4-*N*-alkyl chain length. In addition, we also report preliminary studies toward the intramolecular cyclization of the diphosphate **6** by pyrophosphate bond formation, leading to **8**. The synthesised cyclic diphosphate AICAR analogue **8** can be seen as a new mimic of cADPR, an endogenous metabolite that regulates the Ca^2+^-mobilization via ryanodine receptors (RyR) in various cell types [19,20,21,22,23,24,25,26]. It could also represent a useful molecule for studying the structure-activity relationships of cADPR analogues and exploring the cADPR/RyR Ca^2+^ signalling system.

Our synthetic procedure uses solid support **2** (0.7 mmol/g) (Scheme 1) carrying the *N*-1-(5-hydroxypentyl)inosine bound to a polystyrene resin through a 2',3'-benzylidene linker. Support **2** was prepared according to a procedure previously developed in our laboratories [18,29,30,31] and then converted into the bis-protected diphosphate support **3a** by treatment with bis-(2-cyanoethyl)-*N*,*N*'-diisopropylphosphoramidite [(iPr)_2_NP(OCE)_2_] in the presence of tetrazole, followed by oxidation with *t*-butyl hydroperoxide (*t*-BuOOH). 

The successive treatment of support **3a** with ethanolic NaOH failed to give the desired AICAR diphosphate derivative **5a** through the expected degradation of the pyrimidine ring. We hypothesize that the basic treatment [18] produces the fast loss of the cyanoethyl protecting groups and the negative charges generated on the phosphates prevent the nucleophilic attack of the hydroxyl ion to the electron-deficient C-2 purine carbon, the first step in the six-membered ring degradation [17,30]. This reduced reactivity of C-2 when flanked by a 5'-phosphate confirmes our previous results obtained during the synthesis of ZMP starting from AICAR [18]. Therefore, we used the commercially available bis-trimethylsilylethoxy-*N*,*N*'-diisopropylphosphoramidite [31] [(iPr)_2_NP(OTMSEt)_2_], in which the base-stable TMSEt groups can be removed by fluoride ions or trifluoroacetic acid treatment, for the synthesis of the compounds **4** and **6**. According to this strategy, reaction of **2** with [(iPr)_2_NP(OTMSEt)_2_] in THF, in the presence of tetrazole afforded support **3b** in high yields after the usual phosphite oxidation with *t*-BuOOH. The reaction yields of the bis-phosphorylation steps, leading to **3a** or **3b**, were evaluated analyzing the inosine diphosphate derivative **4** released from a weighted amount of the resins **3a** and **3b**.

In particular, for **3b** the acid treatment assured the complete deprotection of both phosphates as well as the release of the nucleotidic material from the resin. In the case of **3a** ammonia treatment was needed to remove all of the 2-cyanoethyl phosphate protecting groups before the acid treatment. In both cases the bis-phosphorylation yields were in the 90–92% range (from **2**). Spectral data for **4** (from **3b**) were in full agreement with those previously reported [29]. Support **3b** was successively treated with ethanolic NaOH to give the AICAR diphosphate support **5b**. Acid treatment of **5b** with 2% trifluoroacetic acid (TFA) in DCM eventually provided the diphosphate AICAR derivative **6** with 69% yield (from **2**), the structure of which was confirmed by spectroscopic data (see Experimental). ^31^P-NMR data (*δ*_P_ singlets at 1.85 and 1.71) confirmed the presence of two phosphomonoester functions in **6**. Next, the intramolecular cyclization of **6** by pyrophosphate bond formation was addressed. To this end, the diphosphate derivative **6** was converted in 80% yield into 2',3'-isopropylidene derivative **7**, by reaction with dimethoxypropane (DMP) in the presence of catalytic amounts of *p*-toluenesulfonic acid (*p*-TsOH) in DMF. Subsequently, we focused on the cyclization step. In the first attempts, 1-ethyl-3-(3-(dimethylamino)propyl) carbodiimide hydrochloride (EDC) [32] and 1,3*-*dicyclohexylcarbodiimide (DCC) [33,34,35], which have been successfully used in previous instances for the pyrophosphate bond formation, were employed. Disappointingly, in our case both these reagents only gave a complex mixture of products (HPLC profiles) among which no cyclization product could be detected by NMR and mass spectral analyses. We eventually succeeded in obtaining the desired cyclic compound **8** (20% yield) by using 1,1'-carbonyldiimidazole (CDI) (5.0 equiv. in DMF) [36]. Compound **8** was purified by HPLC on a reverse-phase C-18 column and its structure was confirmed by NMR and HRESI-MS data.

## 3. Experimental

### 3.1. General

4-Methoxytrityl chloride resin (MMTCl, 1% divinylbenzene, 200–400 mesh, 1.3 mmol/g loading) was purchased from CBL (Greece). All reagents were obtained from commercial sources (Sigma-Aldrich, Germany) and were used without further purification. The reactions on solid phase were performed using glass columns (10 mm diameter, 100 mm length) with fused-in sintered glass-disc PO (bore of plug 2.5 mm), which were shaken in an orbital shaker, or in round-bottomed flasks, when reactions required high temperatures. ^1^H-NMR spectra were recorded on a Varian Mercury Plus 400 MHz in D_2_O solvent. ^31^P-NMR were recorded on a Varian Unity Inova 500 MHz instrument in D_2_O solvent using 85% H_3_PO_4_ as an external standard (0 ppm). Chemical shifts are reported in parts per million (*δ*) relative to the residual solvent signals: D_2_O 4.80 for ^1^H-NMR. The abbreviations s, bs, d, dd and m stand for singlet, broad singlet, doublet, doublet of doublets and multiplet, respectively. High performance liquid chromatography (HPLC) analyses and purifications were carried out on a Jasco UP-2075 Plus pump equipped with a Jasco UV-2075 Plus UV detector using a 4.8 × 150 mm C-18 reverse-phase column (particle size 5 µm) eluted with a linear gradient of CH_3_CN in 0.1 M triethylammonium bicarbonate (TEAB) buffer, pH = 7.0 (from 0 to 100% in 120 min, flow 1.4 mL/min). UV spectra were recorded on a Jasco V-530 UV spectrophotometer. High Resolution MS spectra were recorded on a Bruker APEX II FT-ICR (9.4 T) mass spectrometer using electrospray ionization (ESI) in negative mode. Analytical TLC analyses were performed using F254 silica gel plates (0.2 mm, Merck). TLC spots were detected under UV light (254 nm).

### 3.2. Preparation of Solid Supports **3b**

Solid support **2** (0.10 g, 0.070 mmol), swollen in dry THF, was treated with (iPr)_2_NP(OTMSEt)_2_ (0.3 g, 0.8 mmol) and tetrazole (0.09 g, 1.3 mmol) in dry THF/DCM (9:1, 2.5 mL) and shaken for 15 h at room temperature. Then the resin was filtered and the solid support was washed with THF (3 × 5 mL), THF/MeOH (1:1, 3 × 5 mL) and MeOH (3 × 5 mL) and finally dried under vacuum. The resin was treated with *t*-BuOOH (5.5 M in decane, 0.4 mL, 2.4 mmol) in THF (2.5 mL) for 2.5 h at room temperature and then washed with THF (3 × 5 mL), THF/MeOH (1:1, 3 × 5 mL), MeOH (3 × 5 mL) and finally dried under vacuum to give **3b** (136 mg). The bis-phosphorylation yield was calculated by detaching the nucleotidic material from a weighted amount of resin **3b** with 2% TFA in DCM for 20 min at room temperature (H_2_O washings collected) and analyzing the crude material by HPLC. From 20 mg of resin **3b**, 6.9 mg of **4** (92% yield from **2**) as bis-triethylammonium salt were obtained.

### 3.3. Preparation of Solid Support **5b**

Resin **3b** (0.10 g, 0.047 mmol) was suspended in a 5 M solution of NaOH in EtOH (2 mL) for 5 h at reflux. The resin was cooled, filtered and washed with EtOH (3 × 5 mL), EtOH/H_2_O (1:1, 3 × 5 mL), EtOH (3 × 5 mL) and then dried under reduced pressure, affording **5b** (99 mg). The reaction yield was calculated by detaching the nucleotidic material from a weighted amount of resin **5b** with 2% TFA in DCM for 20 min at room temperature (H_2_O washings collected) and analyzing the crude by HPLC. From 20 mg of resin **5b**, 4.9 mg of **6** (69% yield from **2**) as bis-triethylammonium salt were obtained.

*5-Amino-1-(β-D-ribofuranosyl)imidazole-4-[N-(5-O-phosphorylpentyl)]carboxamide5'-phosphate* (**6**): White foam, bis-triethylammonium salt; ^1^H-NMR (400 MHz, D_2_O, assignments by HH-COSY) *δ*_H_ 7.58 (s, 1H, 4-H), 5.68 (d, *J* = 6.8 Hz, 1H, 1'-H), 4.66–4.61 (m, 1H, 2'-H), 4.45–4.41 (m, 1H, 3'-H), 4.36–4.31 (m, 1H, 4'-H), 4.13–4.08 (m, 2H, 5'-H_a,b_), 3.93–3.85 (m, 2H, CH_2_O), 3.36 (t, *J* = 6.9 Hz, 2H, CH_2_N), 3.21 (q, *J* = 7.3 Hz, 12H, 6 × CH_2_, triethylammonium), 1.74-1.59 (two overlapped multiplets, 4H, 2 × CH_2_), 1.51-1.41 (m, 2H, CH_2_), 1.29 (t, *J* = 7.3 Hz, 18H, 6 × CH_3_, triethylammonium); ^31^P-NMR (202 MHz, D_2_O) *δ*_P_ 1.85, 1.71 (two singlets); HRESI-MS *m*/*z* 503.0956 ([M-H]^−^, requires 503.0950); UV (H_2_O) *λ*_max_ 266 nm.

### 3.4. Synthesis of Compound **7**

**6** (10 mg, 0.014 mmol) was dissolved in DMF (1.0 mL). *p*-TsOH (catalytic amounts) and DMP (0.017 mL, 0.14 mmol) were added and the mixture was shaken at r.t. for 2 h (TLC monitoring isopropanol/NH_4_OH(*aq*)/H_2_O, 6:3:1). The solvents were evaporated under reduced pressure and the crude was dissolved in 1.0 mL of TEAB 0.1 M (pH = 7.0) solution and then purified by HPLC (see *2.1*, General). The fractions containing the title compound were collected and evaporated under reduced pressure. The target compound was dissolved in H_2_O (3 × 2.0 mL) and was lyophilized three times affording 8.4 mg of pure **7** (80% yield).

*5-Amino-1-(β-D-ribofuranosyl)imidazole-4-[N-(5-O-phosphorylpentyl)]carboxamide-2',3'-O-iso-propylidene 5'-phosphate* (**7**): White foam, bis-triethylammonium salt; ^1^H-NMR (400 MHz, D_2_O, assignments by HH-COSY experiment) *δ*_H_ 7.60 (s, 1H, 4-H), 5.94 (d, *J* = 6.8 Hz, 1H, 1'-H), 5.28–5.23 (m, 1H, 2'-H), 5.13–5.09 (m, 1H, 3'-H), 4.65–4.61 (m, 1H, 4'-H), 4.07–4.01 (m, 2H, 5'-H_a,b_), 3.89–3.82 (m, 2H, CH_2_O), 3.32 (t, *J* = 6.9 Hz, 2H, CH_2_N), 3.16 (q, *J* = 7.3 Hz, 12H, 6 × CH_2_, triethylammonium), 1.68–1.55 (complex signal, 7H, 2 × CH_2_, CH_3_), 1.46–1.37 (complex signal, 5H, CH_2_ and CH_3_), 1.24 (t, *J* = 7.3 Hz, 18H, 6 × CH_3_, triethylammonium); ^31^P-NMR (202 MHz, D_2_O) *δ*_P_ 1.78, 1.67 (two singlets); HRESI-MS *m*/*z* 553.1114 ([M-H]^−^, requires 553.1106); UV (H_2_O) *λ*_max_ 266 nm.

### 3.5. Pyrophosphate Bond Formation: Synthesis of Compound ***8***

Compound **7** (5.0 mg, 0.0067 mmol) was dissolved in DMF (15 mL) and CDI (5.2 mg, 0.033 mmol) was added in one portion. The mixture was shaken at room temperature for 48 h. The solvent was removed under reduced pressure and the crude was dissolved in 1.0 mL of TEAB 0.1 M (pH = 7.0) solution and then purified by HPLC (see General). The fractions containing the title compound were collected and evaporated under reduced pressure. The target compound was dissolved in H_2_O (3 × 2.0 mL) and was lyophilized three times affording 0.84 mg of **8** (20% yield).

*4-N-pentyl-cyclic AICAR-2',3'-O-isopropylidene diphosphate* (**8**): Amorphous solid, triethylammonium salt; ^1^H-NMR (400 MHz, D_2_O, assignments by HH-COSY experiment) *δ*_H_ 7.45 (s, 1H, 4-H), 6.05 (bs, 1H, 1'-H), 5.68–5.62 (m, 1H, 2'-H), 5.43–5.39 (m, 1H, 3'-H), 4.61–4.58 (m, 1H, 4'-H), 3.70–3.65 (m, 2H, 5'-H_a,b_), 3.85–3.79 (m, 2H, CH_2_O), 3.40 (m, 2H, CH_2_N), 3.16 (q, *J* = 7.4 Hz, 6H, 3 × CH_2_, triethylammonium), 1.65–1.58 (complex signal, 7H, 2 × CH_2_, CH_3_), 1.45–1.35 (complex signal, 5H, CH_2_ and CH_3_), 1.24 (t, *J* = 7.4 Hz, 9H, 3 × CH_3_, triethylammonium); ^31^P-NMR (202 MHz, D_2_O) *δ*_P_ −9.5, −10.4; HRESI-MS *m*/*z* 525.1162 ([M-H]^−^, requires 525.1157); UV (H_2_O) *λ*_max_ 265 nm.

## 4. Conclusions

In the present study we have carried out the solid-phase synthesis of a new type of AICAR diphosphate derivative as well as its cyclization to a new AICAR-based mimic of cADPR. Work is in progress to extend our methodology to the synthesis of other analogues of both **6** and **8** by modulating the 4-*N*-alkyl chain length. Biological assays on the synthesised substances are in progress as well.
